# The National Dental Association

**Published:** 1902-09

**Authors:** 


### Editorial.

### THE NATIONAL DENTAL ASSOCIATION.

THIS body met at Niagara Falls, July 28, with the President, Dr. J. A. Libbey, of Pittsburg, in the chair.

The attendance, as usual at Niagara, was large, numbering from four to five hundred, completely crowding the hotels and driving the surplus into other quarters.

The meeting began its first session without any preliminaries, the routine business being given into the hands of the council. The address of the president was full of practical suggestions, which, if carried out, would materially aid the well-being of the organization.

The papers presented were much above the average from a scientific as well as a practical view, and the discussions were well sustained. The general feeling seemed to be that it was one of the best meetings that the National Dental Association has held since the reorganization. The exhibits were large, and during intermissions the rooms were crowded to excess. It is certain, however, that hotel space is not satisfactory either to the exhibitor or dentist. Coming, as the writer did, from the fine exhibit at Asbury Park and the New Jersey State meeting, it seemed defective, but these national meetings cannot have an "Auditorium" to carry around with them. A very large proportion of those in attendance gave more time to an examination of the exhibits than to the meetings. This must be expected at all national and State gatherings, and especially the former. A very large number make sacrifices to attend this meeting, well aware that they can see new and old appliances there, under one roof, that it would be impossible for them to examine otherwise. Many practitioners are for the entire year cut off from the great centres, in isolated regions, and this annual experience brings them in touch with the progress made in the mechanical side of dentistry, giving them an inspiration and help not obtainable otherwise. They feel that this, to them, is so important that they can sacrifice the scientific side of the convention for a more convenient day, which, of course, never comes. It is very evident that dentistry is more and more becoming separated into two distinct divisions, neither of which can be said to be in entire harmony with the other. These two are represented by the man, on the one hand, who loves original research into the unsolved problems, and, on the other, the man who spends otherwise unoccupied time at the bench in his laboratory. The result is a small body of students and readers and a very large class who care little for those things that make a profession, but much for that which enters into the development of an art or a stimulant to trade. It is useless to contend for or against these mental proclivities. They are with us, and must be met and the various tastes provided for, trusting to a future generation to correct these peculiarities and develop a different standard of mental growth. There was a time when the national body refused to have clinical demonstrations at its meetings. The attendance fell off, and the Association was obliged to restore these, and at once the attendance increased.

At the present meeting provision was made for a large number of interesting clinics. These were arranged upon the porticos and lawn of the hotel, thus giving ample room and pleasant surroundings. These comprised operations in minor oral surgery, porcelain inlays, bleaching teeth, orthodontia, crown- and bridge-work, etc., etc. To the writer these clinics seemed to have a special value, as they were so arranged as to cover almost the entire procedures in dental work.

The council of the national body expressed itself positively against conferring the degree of Doctor of Dental Surgery, as proposed by the New York State Dental Society. It was fully recognized that the D.D.S. was national, and that no section of the country had the right to break down, in the slightest, the respect which belonged to this title, or any other secured by years of consistent undergraduate work.

The Association next year will go South, and there can be no doubt but that our Southern brethren will strive to make it a memorable occasion. Every effort should be made in the North to aid them in securing a satisfactory meeting. Much can be accomplished in the next twelve months by systematic efforts in the sections, but to do this effectively it must begin at once. The evidence of improvement in the work of these subordinate bodies was very evident at this meeting, but a more strict censorship is still desirable. The ideal of the writer will not, however, be reached under present methods of organization. It needs no prophetic knowledge to say that, when this present century is well advanced and the graduates of a four years curriculum are in the ascendant, the present crude efforts will have had their day and be abandoned for more effectual and thorough work. The question will not then be asked, What does a man believe? but, What does he know? The man of facts will take the foremost place, and the man of imagination will have been retired to the professional background, and when this time arrives there will be a dental profession worthy to be named with the honorable callings of the world.

